# Multiple Transceptors for Macro- and Micro-Nutrients Control Diverse Cellular Properties Through the PKA Pathway in Yeast: A Paradigm for the Rapidly Expanding World of Eukaryotic Nutrient Transceptors Up to Those in Human Cells

**DOI:** 10.3389/fphar.2018.00191

**Published:** 2018-03-13

**Authors:** Fenella Steyfkens, Zhiqiang Zhang, Griet Van Zeebroeck, Johan M. Thevelein

**Affiliations:** ^1^Laboratory of Molecular Cell Biology, Institute of Botany and Microbiology, KU Leuven, Leuven, Belgium; ^2^Center for Microbiology, VIB, Flanders, Belgium

**Keywords:** *Saccharomyces cerevisiae*, transceptor, nutrient sensing, nutrient signaling, protein kinase A, nutrient starvation, transporter induction, evolution

## Abstract

The nutrient composition of the medium has dramatic effects on many cellular properties in the yeast *Saccharomyces cerevisiae*. In addition to the well-known specific responses to starvation for an essential nutrient, like nitrogen or phosphate, the presence of fermentable sugar or a respirative carbon source leads to predominance of fermentation or respiration, respectively. Fermenting and respiring cells also show strong differences in other properties, like storage carbohydrate levels, general stress tolerance and cellular growth rate. However, the main glucose repression pathway, which controls the switch between respiration and fermentation, is not involved in control of these properties. They are controlled by the protein kinase A (PKA) pathway. Addition of glucose to respiring yeast cells triggers cAMP synthesis, activation of PKA and rapid modification of its targets, like storage carbohydrate levels, general stress tolerance and growth rate. However, starvation of fermenting cells in a glucose medium for any essential macro- or micro-nutrient counteracts this effect, leading to downregulation of PKA and its targets concomitant with growth arrest and entrance into G0. Re-addition of the lacking nutrient triggers rapid activation of the PKA pathway, without involvement of cAMP as second messenger. Investigation of the sensing mechanism has revealed that the specific high-affinity nutrient transporter(s) induced during starvation function as transporter-receptors or transceptors for rapid activation of PKA upon re-addition of the missing substrate. In this way, transceptors have been identified for amino acids, ammonium, phosphate, sulfate, iron, and zinc. We propose a hypothesis for regulation of PKA activity by nutrient transceptors to serve as a conceptual framework for future experimentation. Many properties of transceptors appear to be similar to those of classical receptors and nutrient transceptors may constitute intermediate forms in the development of receptors from nutrient transporters during evolution. The nutrient-sensing transceptor system in yeast for activation of the PKA pathway has served as a paradigm for similar studies on candidate nutrient transceptors in other eukaryotes and we succinctly discuss the many examples of transceptors that have already been documented in other yeast species, filamentous fungi, plants, and animals, including the examples in human cells.

## Biological Systems that Led to the Discovery of Transceptors

Cells of the yeast *Saccharomyces cerevisiae* growing in a complete nutrient medium with a fermentable carbon source display a range of phenotypes indicating that the activity of PKA is high, while the opposite is true for cells growing on a non-fermentable carbon source or for stationary phase cells (Lillie and Pringle, 1980). Characteristics for the low PKA phenotype are reduced expression of ribosomal protein genes, low growth rate, upregulated stress tolerance mechanisms, such as higher expression of heat shock protein genes, higher accumulation of reserve carbohydrates like trehalose and glycogen, and stronger, more lyticase-tolerant cell walls (Thevelein and de Winde, 1999).

### Glucose

Early observations have shown that addition of glucose to ascospores or glucose-derepressed vegetative cells of *S. cerevisiae*, causes within minutes, a spike in the cAMP level followed by a transient activation of the trehalase enzyme, a target of PKA. The activation of trehalase causes mobilization of trehalose, which is hydrolyzed into two glucose molecules (van der Plaat, 1974; Thevelein et al., 1982). Later, it was found that after a period of inactivation, in both the ascospores and the vegetative cells, the observed spike in cAMP as well as the transient activation of trehalase can be re-induced, although not to a full extent (Thevelein and Jones, 1983; Thevelein, 1984). The activation of trehalase is commonly used as a direct PKA read-out, since it has been shown to be directly phosphorylated by PKA both *in vitro* and *in vivo* (Schepers et al., 2012). The mechanisms by which glucose causes activation of the cAMP-PKA pathway have subsequently been elucidated in great detail (Conrad et al., 2014).

Glucose and sucrose act as ligands for the GPCR, Gpr1 (Kraakman et al., 1999; Lemaire et al., 2004). The corresponding G-protein signal transmitter, Gpa2, passes the signal on to adenylate cyclase, Cyr1. Similar to mammalian cells, adenylate cyclase synthesizes cAMP from ATP, thereby activating the central heterotetrameric signaling hub PKA. Compared to the situation in mammalian cells, *S. cerevisiae* Gpa2 does not constitute a traditional G_α_-protein as it does not associate with a classical G_βγ_-subunit, in spite of its clear sequence similarity with classical G_α_-proteins. The kelch-repeat homologs, Krh1 and Krh2, which physically bind to Gpa2, have a predicted seven-bladed β-propeller structure and were initially proposed to act as G_β_-mimics for Gpa2 (Harashima and Heitman, 2002). Later on, however, they were shown to function as direct inhibitors of PKA. They act by stimulating the binding between the catalytic and regulatory subunits of PKA and are inhibited in response to activation of Gpa2 by glucose, thus bypassing adenylate cyclase for additional activation of PKA (Peeters et al., 2006). This is achieved through inhibition by Krh1/2 of PKA mediated phosphorylation of Bcy1 (Budhwar et al., 2011). Gpa2 was also shown to bind Asc1, which inhibits its GDP/GTP exchange and was proposed to act as an alternative G_β_ subunit (Zeller et al., 2007).

**FIGURE 1:**
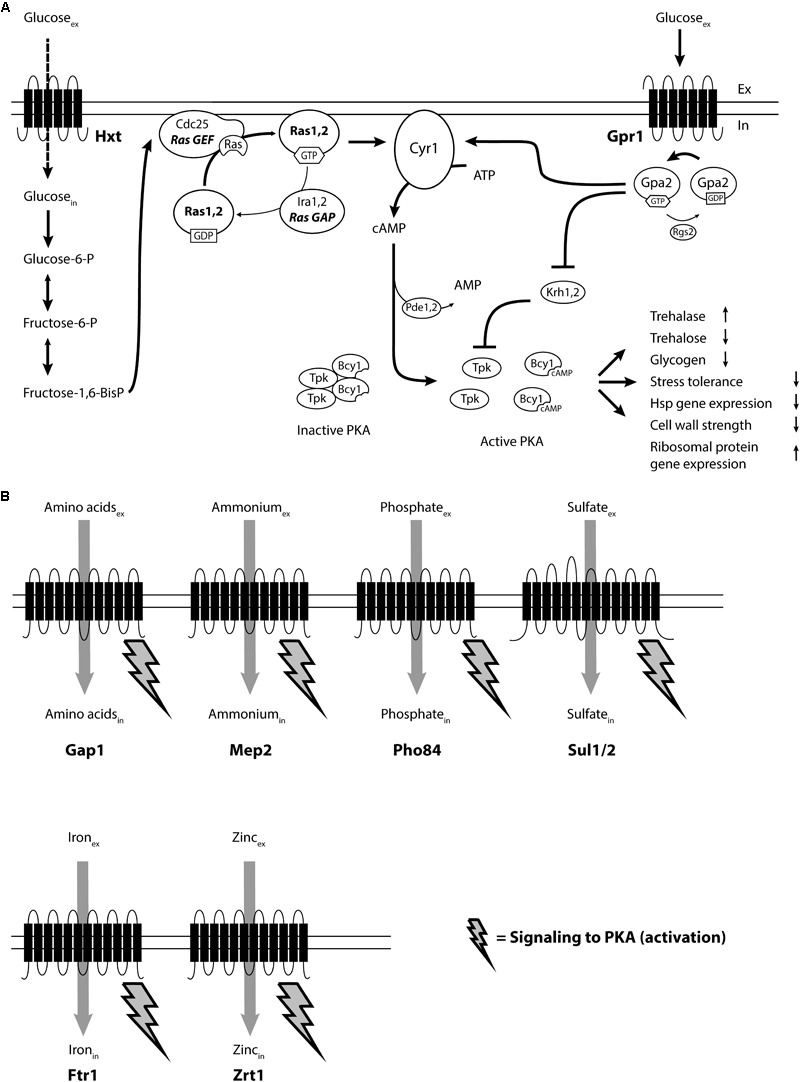
Overview of receptor- and transceptor-mediated nutrient activation of PKA in the yeast *Saccharomyces cerevisiae*. **(A)** Glucose binding to the G-protein-coupled receptor Gpr1, together with intracellular activation of the Ras proteins triggers activation of adenylate cyclase (Cyr1), to synthesize cAMP. The rapid increase in cAMP releases the Tpk catalytic subunits from the Bcy1 regulatory subunits of PKA, resulting in activation or downregulation of the many downstream targets of PKA: trehalase activity, trehalose and glycogen levels, stress tolerance, heat shock protein gene expression, cell wall strength, ribosomal protein gene expression and many other targets. **(B)** Addition of other essential nutrients, in the presence of a fermentable carbon source, to appropriately starved yeast cells triggers activation of the catalytic subunits of PKA, without any increase in the cAMP level. Nutrient sensing happens with a specific nutrient transceptor, different for each nutrient and previously known as substrate starvation-induced high-affinity transporters. How the signal is transmitted from the nutrient transceptor to PKA is under investigation.

The GPCR-based extracellular glucose-sensing mechanism, however, is unable to activate adenylate cyclase and stimulate cAMP synthesis as long as adenylate cyclase is not at the same time activated by the Ras proteins. Remarkably, the latter stimulation requires transport of glucose into the cell as well as its partial breakdown in glycolysis to generate fructose-1,6-bisphosphate, which activates Ras through direct stimulation of its guanine nucleotide exchange factor, Cdc25 (Peeters et al., 2017). Glucose activation of cAMP synthesis in yeast thus requires the interplay of an extracellular receptor-based glucose-sensing system with an intracellular glucose-sensing system based on partial metabolic breakdown of the sugar. It was the first example of such a combined sensing system ever discovered. A schematic overview of the two mechanisms for combined activation of the cAMP-PKA pathway is shown **Figure 1**.

It remains unclear whether the high-affinity glucose transporters Hxt6 and Hxt7, that are induced in medium lacking glucose, also play a role in glucose-induced activation of the PKA pathway. A yeast mutant, *lcr1* = *Cyr1^K1876M^*, has been identified that completely lacked the glucose-induced cAMP signal, but still displayed strong activation of the PKA target trehalase (Vanhalewyn et al., 1999). This indicates that an alternative pathway, not involving cAMP as second messenger, must be responsible. This would fit with the action mechanism of the transceptors described below, but alternatively the kelch-repeat protein bypass pathway of adenylate cyclase could also be responsible for this part of glucose-induced activation, since it allows activation of PKA without cAMP signaling (Peeters et al., 2006, 2007).

### Other Nutrients

The first hint for an alternative nutrient signaling mechanism to PKA was obtained with glucose-derepressed (respiring) cells in which the cAMP-PKA pathway had first been activated by glucose, as evidenced by transient activation of trehalase, and to which a nitrogen source was added to support growth initiation by the cells. Interestingly, addition of the nitrogen source caused an immediate reactivation of trehalase, without any change in the cAMP level (Thevelein and Beullens, 1985). This indicated the presence of an alternative mechanism for activation of PKA by nitrogen sources. To render the reactivation of trehalase more pronounced and more reproducible, the incubation period in glucose medium was prolonged to 24 h, so that the cells actually arrested growth due to nitrogen starvation and accumulated in the stationary phase, G_0_ (Hirimburegama et al., 1992). As a result, this nitrogen-induced activation of trehalase was associated with induction of growth and progression over the ‘START’ point in the G1 phase of the cell cycle.

By using temperature-sensitive mutants in cAMP synthesis at permissive and restrictive temperature it was confirmed that nitrogen-, phosphate-, and sulfate-induced activation of trehalase do not use cAMP as second messenger, although maintenance of a basal level of cAMP is required for full activation (Hirimburegama et al., 1992). Because of the dual requirement of a fermentable carbon source and a complete growth medium, the pathway involved in maintaining high PKA activity during growth on a fermentable carbon source, has been called the FGM induced pathway (Thevelein, 1994). Although the precise relationship between the cAMP-PKA pathway and the FGM pathway is unclear, both seem to be required for sustained activation of PKA in fermentatively growing cells.

Recently, the concept of non-cAMP mediated activation of PKA through the FGM pathway has been expanded from the macro-nutrients, nitrogen, phosphate, and sulfate, to the micro-nutrients iron and zinc (Schothorst et al., 2017). In each of these cases, re-addition of the missing nutrient to specifically starved cells is sensed by high-affinity transporters that are strongly induced upon starvation for their substrate and were shown to have an additional receptor function. Because of this dual function, these membrane proteins were called ‘transceptors’ (Holsbeeks et al., 2004). Signaling is triggered by binding of a substrate to the transceptor and causes rapid activation of PKA concomitant with the upstart of growth and progression over the ‘START’ point in the cell cycle. Finally, for all these transceptors substrate-induced internalization has been reported, which either strongly reduces or nearly eliminates their presence during subsequent exponential growth. The different nutrient transceptors known in *S. cerevisiae* will be discussed in the next section. An overview of the different nutrient transceptors identified in *S. cerevisiae* and their role in activation of PKA in concert with the cAMP-PKA pathway is shown in **Figure 1**.

## Specific Nutrient Transceptors in *S. cerevisiae*

Proving an additional nutrient sensing property of a transporter is not straightforward and requires several levels of evidence (1) excluding the involvement of intracellular sensing and thus also of other transporters that can take up the relevant substrate, (2) identification of non-transported competitive or non-competitive inhibitors with agonist signaling function, (3) transport-deficient versions of the transceptor that have retained the signaling function, for instance by mutating the residues involved in proton-coupling required for symport of the substrate (Schothorst et al., 2017), and (4) identification of the transceptor signaling mechanism would be the final confirmation that the transceptor functions both as a nutrient transporter and a nutrient receptor.

### The Amino Acid Transceptor Gap1

The general amino acid permease Gap1 has been shown to be a transceptor responsible for amino acid-induced activation of PKA targets (Donaton et al., 2003). Under nitrogen starvation or during growth on a poor nitrogen source, expression of *GAP1* is strongly enhanced and the protein is present in high quantity at the plasma membrane (Magasanik and Kaiser, 2002). Amino acid induced activation of PKA, as inferred from the effects on its downstream targets, is abolished in a *gap1*Δ strain, indicating that Gap1 is somehow required for the activation. Deletion of *GAP1* abolishes amino-acid induced activation of trehalase, mobilization of trehalose and glycogen, loss of heat-shock tolerance, repression of heat shock protein genes and genes encoding other stress-protection proteins as well as induction of ribosomal protein genes (Donaton et al., 2003).

High concentrations of L-citrulline, which are taken up by other transporters in a *gap1*Δ strain, do not trigger activation of PKA targets, contradicting intracellular sensing and pointing to Gap1 itself as the amino acid sensor. Activation of PKA targets was also observed upon addition of non-metabolizable D-amino acids, which can be transported by Gap1, contradicting the involvement of metabolism in triggering signaling. Similarly, L-citrulline induced activation of PKA targets was observed in a wild type strain lacking the first enzyme of L-citrulline metabolism, argininosuccinate synthase, which again excluded a role for metabolism (Donaton et al., 2003). Later, identification of a non-transported signaling agonist, L-Leu-Gly, conclusively established Gap1 as a transceptor, and showed that its sensing role is independent of complete transport of the substrate through the transporter. SCAM (Substituted Cysteine Accessibility Method) analysis showed that transport and signaling use the same substrate binding site in Gap1 (Van Zeebroeck et al., 2009). However, the later identification of a competitive transport inhibitor, L-Asp-γ-L-Phe, that failed to act as a signaling agonist, indicates that binding of a compound into the binding site is not enough to trigger signaling. Hence, activation of signaling by substrate binding to a transceptor requires induction of a specific conformational change in the transceptor. This was further confirmed by the later discovery of three amino acids, L-lysine, L-histidine and L-tryptophan, that are transported by Gap1 with similar rates as the other amino acid substrates, but without triggering signaling (Van Zeebroeck et al., 2014). This also supports that substrates interact with different amino acid residues during their passage through a transporter and are therefore able to trigger different intermediate conformations, only some of which can interact with downstream regulatory proteins in a proper way so as to initiate the signaling event.

### The Ammonium Transceptor Mep2

Similar to amino acid addition, also the addition of ammonium to nitrogen-starved cells triggers rapid activation of PKA, as inferred from the effect on multiple read-outs (Van Nuland et al., 2006). As observed for amino acid activation, ammonium-induced activation of PKA is also not mediated by an increase in cAMP. Three channel-type ammonium transporters exist in yeast, the permeases Mep1-3. The expression of all three *MEP* genes is induced under nitrogen starvation, with *MEP2* being the most strongly induced permease (Marini et al., 1997). Ammonium-induced effects on PKA targets in nitrogen-starved cells are abolished in a triple *MEP* deletion strain, indicating the requirement of the Mep proteins. Experiments with strains in which only one *MEP* gene was expressed in the triple deletion strain showed that ammonium-induced signaling to PKA is mainly dependent on Mep2, while Mep1 also supports signaling, but to a lower extend (Van Nuland et al., 2006).

Abolishing incorporation of ammonium into metabolism did not prevent ammonium-induced activation of PKA, while the addition of low concentrations of the non-metabolizable ammonium-analog methylamine in a strain expressing only Mep2 also results in activation. High concentrations of methylamine can diffuse through the plasma membrane in a triple *MEP* deletion strain but this does not trigger activation of the PKA target trehalase. These observations contradict a role for metabolism as well as intracellular sensing in ammonium-induced signaling to PKA (Van Nuland et al., 2006). Moreover, specific mutations have been identified in Mep2 that differentially affect its transport and signaling function for both ammonium and the transported, non-metabolizable signaling agonist methylamine, further establishing Mep2 as a transceptor. Mep2 is regulated by phosphorylation. Under nitrogen-replete conditions Mep2 is not phosphorylated and present in an inactive, closed conformation. Phosphorylation triggers major changes in conformation, suggesting that phosphorylation may enable the sensing function of Mep2 (van den Berg et al., 2016).

The Mep2 ammonium transceptor had previously been shown to function also in control of pseudohyphal growth in diploid *S. cerevisiae* cells. Deletion of *MEP2*, but not *MEP1* or *MEP3*, abolishes pseudohyphal growth induction upon nitrogen deprivation. This defect can be overcome by overactivation of the cAMP-PKA pathway in a *mep2*Δ*/mep2*Δ strain (Lorenz and Heitman, 1998). These observations suggest that Mep2 also connects to the PKA pathway to control morphogenesis in yeast.

### The Phosphate Transceptor Pho84

The high-affinity phosphate transporter, Pho84, has been identified as the main transceptor responsible for phosphate-induced activation of the PKA targets in phosphate-starved cells (Giots et al., 2003; Popova et al., 2010). Deletion of *PHO84* prevented phosphate activation of all PKA targets investigated. Under conditions of phosphate starvation, expression of the high-affinity phosphate transporters Pho84 and Pho89 is strongly induced (Martinez et al., 1998). In a *pho84*Δ strain, phosphate-induced activation of the PKA target trehalase as well as phosphate uptake are strongly reduced. Pho89 did not seem to play a role in phosphate signaling. In subsequent work, the transport and receptor functions could be separated in different ways. First by identification of the non-transported, competitive phosphate-transport inhibitor Gly3P as a Pho84-dependent signaling agonist (Popova et al., 2010). The discovery of this molecule showed that Pho84 could act as a genuine receptor and that the ligand actually uses the same substrate-binding site as is used for the transport of the regular substrate, phosphate. Gly3P is transported into the cells by the Git1 and Pho91 carriers, but in this case there is no signaling to PKA (Popova et al., 2010). Second, by insertion of specific point mutations in the putative H^+^-binding residues (Samyn et al., 2012, 2016). This abolishes the transport of phosphate through the Pho84 transporter, but apparently still allows proper binding of phosphate into the substrate-binding pocket as well as proper induction of the conformational change that triggers signaling to the PKA pathway. The involvement of metabolism has been excluded by identification of the non-metabolizable analog arsenate, which also acts as a signaling agonist when it is transported through Pho84. The observation that phosphonoacetic acid, a competitive inhibitor of Pho84 transport, does not act as a signaling agonist, indicates that mere binding of a ligand to the transporter is not enough to trigger signaling.

### The Sulfate Transceptors Sul1 and Sul2

As mentioned earlier, the addition of sulfate to sulfate-starved, glucose-repressed cells also activates the PKA pathway as shown by the rapid effects on all downstream targets investigated and using a mechanism that apparently does not involve cAMP as second messenger (Hirimburegama et al., 1992; Kankipati et al., 2015). In this case, it is also associated with induction of fermentable growth, during which high PKA activity is further maintained by the FGM pathway. Sulfate-induced activation of the PKA targets requires at least one of the high-affinity sulfate transporters Sul1 or Sul2 (Kankipati et al., 2015). These transporters are strongly upregulated under conditions of sulfur-starvation (Boer et al., 2003).

The identification of D-glucosamine 2-sulfate as a non-transported signaling agonist both for Sul1 and Sul2, clearly indicates that the substrate does not have to be transported into the cytosol for activation of the signaling function of Sul1 and Sul2. Hence, it clearly highlights the additional signaling function of these permeases (Kankipati et al., 2015). Their role as transceptor has been confirmed further by mutational analysis of residues presumably involved in proton-coupling during symport. This resulted in mutant Sul1 and Sul2 proteins with abolished transport, but with maintenance of their normal signaling function to the PKA pathway (Kankipati et al., 2015). These results clearly showed their capability of functioning both as a transporter and a receptor and thus established *S. cerevisiae* Sul1 and Sul2 as the first sulfate transceptors identified.

### Transceptors for Micro-Nutrients: Ftr1 for Iron and Zrt1 for Zinc

Similar to what happens with macro-nutrients, starvation for micro-nutrients, such as iron and zinc, causes growth arrest in the G_1_ phase, followed by entry into the stationary phase G0, which is characterized by a low PKA phenotype. Re-addition of iron or zinc to iron- or zinc-starved cells, respectively, triggers the same rapid effects on PKA targets, as the re-addition of a macro-nutrient to cells starved for that nutrient. Expression of the high-affinity transporters Ftr1 for iron (Stearman et al., 1996) and Zrt1 for zinc (Zhao and Eide, 1996) is strongly enhanced upon iron or zinc starvation, respectively, and they are stably inserted at the plasma membrane. Iron-induced activation of the PKA target trehalase is abolished in an *ftr1*Δ strain or upon deletion of *FET3*, which results in mislocalization of Ftr1. This indicates that Ftr1 is required in some way for signaling to the PKA pathway. However, deletion of Ftr1 does not abolish uptake of 100 μM FeCl_2_ or FeCl_3_, indicating that activation of the PKA pathway is not triggered by intracellular sensing of iron and must originate from Ftr1. These results established a role for Ftr1 as transceptor (Schothorst et al., 2017).

A similar situation pertains for zinc-induced activation of the PKA pathway through Zrt1. Deletion of *ZRT1* largely abolishes zinc-induced activation of the PKA target trehalase, both at low and high concentrations of zinc. However, high concentrations of zinc are still taken up to a significant extent into the cells in a *zrt1*Δ strain, while they are unable to trigger activation of the PKA pathway. This indicates that zinc-induced PKA activation also does not originate from an intracellular sensing system but must originate from the Zrt1 transporter. The transport rate of high concentrations of zinc in a *zrt1*Δ strain was actually comparable to that of low concentrations of zinc in a wild type strain. Only the latter were able to trigger activation of the PKA target trehalase indicating that Zrt1 also acts as a high-affinity sensor for zinc in addition to its high-affinity transport capacity. Moreover, through mutational analysis, Zrt1^E226Q^ was obtained, in which zinc uptake was abolished with 70% while signaling was not affected. This partial uncoupling of both functions provides strong additional evidence for the dual function of Zrt1 as transporter and receptor (Schothorst et al., 2017).

### Other Possible Transceptors in *S. cerevisiae*

There are several clear similarities between the known nutrient transceptors in *S. cerevisiae*. They are all high-affinity transporters with strongly induced expression upon starvation for their substrate. They are present in a highly active and stable form at the plasma membrane when starved for their substrate. Upon addition of their substrate they are completely or partially internalized by endocytosis, likely preceded in all cases by ubiquitination, so as to reach the level that is also present in exponentially growing cells. They have a similar structure, with multiple transmembrane domains typical of a nutrient transporter. The only exception is the Mep2 ammonium transporter, which is a channel-type protein. Deprivation of nitrogen, phosphate or sulfate leads to upregulation of other transporters for various nitrogen, phosphate or sulfur containing compounds, respectively, that can serve as nutrients for yeast cells. These may also exert a transceptor function upon addition of their substrate to appropriately starved cells.

There are also other essential nutrients for which starvation leads to upregulation of a high-affinity transporter, such as copper and magnesium. Possible candidate transceptors that fulfill the characteristics common with the other transceptors, are the copper transporter Ctr1, the magnesium transporter Alr1 and the broad-specificity metal ion transporter Smf1 (Ooi et al., 1996; Graschopf et al., 2001; Eguez et al., 2004). Preliminary results indicate that copper can trigger activation of the PKA target trehalase in copper-starved cells. This appears to occur independently of Ctr1 (Schothorst, 2016). Likewise, re-addition of adenine or uracil to cells starved for the respective compound, elicits activation of the PKA target trehalase. Also in this case, the possible involvement of a transceptor remains unclear (Schothorst, 2016).

### Similarities with Classical Receptors

Classical receptors, such as those of the GPCR family, may be derived in evolution from nutrient transceptors (Thevelein and Voordeckers, 2009). It has been pointed out previously that most neurotransmitters are nutrients or nutrient-like molecules and that their receptors may thus be derived from nutrient transporters (Boyd, 1979). Interestingly, evidence has been reported that glutamate signaling in neuronal astrocytes may not only be mediated by dedicated glutamate receptors, but also by glutamate transporters functioning as transceptors (Abe and Saito, 2001). Hence, it is attractive to consider possible similarities between nutrient transceptors and classical receptors in order to evaluate a possible common evolutionary origin and to deduce possible hypotheses on functionality that may form a fruitful basis for further experimental analysis.

#### Desensitization by Substrate/Ligand Induced Endocytosis

One striking similarity between nutrient transceptors and GPCRs is their capacity to undergo substrate/ligand induced internalization by endocytosis. Ligand-induced internalization of GPCRs is considered one of the mechanisms used to desensitize mammalian cells and prevent overactivation of the downstream signaling pathway (Bohm et al., 1997; Sorkin and Von Zastrow, 2002). They undergo feedback regulation and receptor endocytosis to keep a balance between receptor signaling and desensitization (Ferguson and Caron, 1998; Irannejad et al., 2015). The agonist-binding stimulated desensitization is regulated by G-protein coupled receptor kinases (GRKs) and β-arrestins, leading to GPCR internalization, endocytosis, intracellular trafficking and/or resensitization (Tsuga et al., 1994; Ferguson et al., 1996; Oakley et al., 1999; Ferguson, 2001).

Endocytosis of high-affinity nutrient transporters has been interpreted previously as degradation of superfluous proteins and/or prevention of ‘substrate-induced cell death’ due to overaccumulation of substrate and/or derivatives in the cytosol (Lauwers et al., 2010). However, nutrient transceptors may also undergo endocytosis to evoke desensitization, similar to classical receptors (Kriel et al., 2011). The nutrient transceptors discovered in yeast activate the PKA pathway and it is well known that overactivation of the PKA pathway causes multiple cellular defects (Rubio-Texeira et al., 2010). The nutrient transceptors are ubiquitinated and internalized for subsequent degradation in the vacuole. Substrate-induced down-regulation of the Gap1 amino acid transceptor has been extensively studied (Chen and Kaiser, 2002; Lauwers et al., 2010). Gap1 ubiquitination requires Rsp5, a ubiquitin ligase, which is conserved in higher eukaryotic cells, and its adaptors Bul1,2 (De Craene et al., 2001; Rotin and Kumar, 2009). Transceptor signaling may also affect the ubiquitin ligase and/or the adaptor proteins, as is well known for receptors (Polo, 2012), but no evidence in this respect has yet been obtained. The initiation mechanism of Gap1 transceptor ubiquitination is still not well understood. It has been proposed that intracellular amino acids promote Gap1 ubiquitination via TORC1/Npr1/14-3-3-dependent control of the arrestin-like adaptors, Bul1,2 (Merhi and Andre, 2012). Similar to receptor desensitization, multiple examples of arrestin-like adaptor proteins (called Arrestin-Related Trafficking adaptors or ARTs) involved in nutrient transceptor and transporter internalization in yeast have now been reported (Lin et al., 2008; Nikko et al., 2008; Nikko and Pelham, 2009; Hatakeyama et al., 2010; O’Donnell et al., 2010; Becuwe et al., 2012; MacGurn et al., 2012; Karachaliou et al., 2013; Nakashima et al., 2014; Guiney et al., 2016; Gournas et al., 2017; Hovsepian et al., 2017; Talaia et al., 2017). Also for the mammalian amino acid transceptor SNAT2 regulation by ubiquitination of endocytosis and proteasomal degradation has been reported (Hyde et al., 2005; Nardi et al., 2015).

#### Endocytosis Triggered by Non-transported Ligands

Evidence has been presented that extracellular non-transported nutrient analogs can trigger endocytosis of the Gap1 amino acid transceptor, creating a similar situation as in ligand-induced receptor internalization. Unexpectedly, evidence was also obtained that an extracellular non-transported nutrient analog L-Asp-gamma-L-Phe, which acts as a competitive inhibitor of Gap1 transport and thus appears to bind into the amino acid binding site, triggers ubiquitination without subsequent endocytosis. Further work also revealed that the transported amino acid L-lysine triggers ubiquitination without subsequent endocytosis and that certain amino acids in concentrations below their Km, are transported without triggering endocytosis (Van Zeebroeck et al., 2014). Hence, ubiquitination is not enough to trigger endocytosis. The latter appears to require an additional event, possibly a specific conformational change in the transporter. Evidence has been obtained that substrate-induced endocytosis of Gap1 and Can1 (arginine permease) requires transition of the permease to a conformational state preceding substrate release into the cell. This transient conformation must be stable enough for the permease to undergo efficient downregulation (Ghaddar et al., 2014). Transport-elicited ubiquitination of Can1 is promoted by transition to an inward-facing state. This conformational change unveils a region of the N-terminal cytosolic tail targeted by the Art1 α-arrestin, which leads to endocytosis (Gournas et al., 2017). The presence of alternating conformational states and binding sites in nutrient transporters (Diallinas, 2014) is in agreement with the view that transport-associated conformational changes can unlock novel binding domains on the intracellular parts of the transceptor.

#### Signaling Triggered by Non-transported Ligands

Similarity between nutrient transceptors and classical receptors is also shown by the discovery of non-transported nutrient analogs that can trigger signaling by direct interaction with the transceptor substrate-binding site: L-Leu-Gly for Gap1 (Van Zeebroeck et al., 2009), Gly3P and other organic phosphate esters for Pho84 (Popova et al., 2010) and D-glucosamine 2-sulfate for Sul1,2 (Kankipati et al., 2015). This indicates that complete transport and metabolism of the nutrient substrate are not essential for triggering transceptor signaling. Nutrient transceptors therefore resemble receptors also in the sense that they can activate the intracellular signaling pathway upon interaction with a ligand that remains outside the cell.

As found for nutrient transport and nutrient-induced endocytosis, the relationship between nutrient transport and nutrient-induced signaling in transceptors is also not straightforward. Initial results indicated a close relationship for Gap1 between transport and signaling, which was difficult to separate (Donaton et al., 2003; Van Zeebroeck et al., 2009). However, later results identified non-transported signaling agonists, but also regular transported amino acids (L-lysine, L-histidine, and L-tryptophan) which did not trigger signaling and showed that other transported amino acids do not trigger signaling in concentrations below their Km (Van Zeebroeck et al., 2014). These observations contradict proposals in the literature of ‘activity-dependent transporter regulation,’ in which complete transport of the nutrient by the transporter is seen as an obligatory requirement for a subsequent event of transporter regulation (Jennings and Cui, 2012; Savir et al., 2017).

#### Substrate-Induced Conformational Changes

The precise mechanisms involved in transceptor initiation of ubiquitination, endocytosis and signaling, and their relationship with transport through the translocation channel, thus remain rather unclear. Every specific event happening in a transceptor upon binding of the substrate appears to require its own specific conformational change in order to allow the event to proceed. Binding of substrates into the binding pocket used for entry into the translocation channel probably triggers divergent substrate-dependent conformational changes, generating different downstream processes such as transport, signaling, ubiquitination and endocytosis. The results support the concept that different substrates bind to partially overlapping binding sites in the same general substrate-binding pocket of Gap1, triggering divergent conformations, resulting in distinct conformation-induced downstream processes (Van Zeebroeck et al., 2014; Diallinas, 2017).

#### Intracellular Signaling From Endosomes

It is well known that GPCRs can continue signaling after their internalization into endosomes (Rajagopalan, 2010; Irannejad et al., 2013; Irannejad and von Zastrow, 2014). Delayed recycling of the beta2-adrenergic receptor results in a concomitant increase in beta2-adrenergic-dependent endosomal signaling (Tian et al., 2016). A similar observation was made with the Gap1 amino acid transceptor, for which three specific γ-glutamyl dipeptides have been identified that cause persistent activation of PKA, prevent Gap1 vacuolar sorting and cause Gap1 accumulation in endosomes. This suggests that the Gap1 transceptor can continue signaling upon internalization into endosomes (Rubio-Texeira et al., 2012).

#### Recycling to the Plasma Membrane After Endocytosis

Another similarity between receptors and transceptors is that they can be recycled after endocytosis rather than being sent to the lysosome/vacuole for degradation. Several classical examples of receptor recycling have been studied in great detail (Grant and Donaldson, 2009). Plasma membrane protein recycling appears to be mediated by an evolving endosomal network able to segregate proteins for different destinations (Bonifacino and Rojas, 2006). Nutrient transceptor recycling has been shown in yeast for Gap1 (Rubio-Texeira and Kaiser, 2006) and Ftr1 (Strochlic et al., 2007; Shi et al., 2011). While ubiquitination is required for transceptor endocytosis, deubiquitination is likely required for recycling to the plasma membrane, similar to the situation in receptors (Kommaddi et al., 2015) and already observed for the yeast Jen1 monocarboxylate transporter (Becuwe and Leon, 2014). For Ftr1, several proteins involved in the recycling mechanism have been identified. When iron is not available, Fet3-Ftr1 is maintained on the plasma membrane via an endocytic recycling pathway requiring the sorting nexin Grd19/Snx3, the pentameric retromer complex, and the Ypt6 Golgi Rab GTPase module. A recycling signal in Ftr1 was identified and found to bind directly to Grd19/Snx3 (Strochlic et al., 2007). The arginine transporter Can1 is recycled back to the cell surface via two independent pathways mediated by the sorting nexins Snx4/41/42 and the retromer complex, respectively. Two novel WD40-domain endosomal recycling proteins, Ere1 and Ere2, that function in the retromer pathway have been identified (Shi et al., 2011). In mammalian cells, a well-known example of transporter recycling is the insulin-induced fusion with the plasma membrane of vesicles containing a reserve pool of GLUT4 glucose transporter (Hoffman and Elmendorf, 2011). Whether GLUT4 may act itself as a transceptor for glucose sensing is not clear (Reno et al., 2017). On the other hand, also for the mammalian amino acid SNAT2 transceptor, accumulation in endosomes and insulin-induced fusion with the plasma membrane causing increased SNAT2 uptake activity has been reported (Hyde et al., 2002).

#### Biased Agonism

It is now well established that binding of different agonists to GPCRs can trigger distinct active conformational changes causing downstream signaling to different pathways by interacting with specific effector systems (Reiter et al., 2012; Wisler et al., 2014). Up to now, no evidence for this so-called ‘biased agonism’ has been reported for nutrient transceptors, but its existence cannot be excluded. Recent evidence indicates that the yeast high-affinity nutrient transceptors that signal to the PKA pathway also interact with other regulatory proteins, suggesting that different nutrient substrates may trigger to some extent different intracellular signaling events.

## Physiological Relevance

### Nutrients Act as Hormones on Cells

The discovery of the glucose-sensing GPCR, Gpr1, in yeast created the novel concept that nutrients could act as hormones on cells and actually use the same mechanisms for detection and signaling as used by hormones in higher cells. Indeed, the glucose-sensing GPCR system in yeast triggers activation of PKA through rapid production of the second messenger cAMP, similar to the action mechanism of for instance the epinephrine-sensing beta-adrenergic receptor in mammalian cells (Levitzki, 1988; Gancedo, 2013). In both cases, activation of PKA results in a downstream phosphorylation cascade rapidly adjusting cellular energy metabolism, e.g., by triggering mobilization of storage carbohydrates (Scott, 1991; Tasken et al., 1997). The glucose response in yeast and the epinephrine response in mammalian cells do not only use the same glucose sensing system, but also affect similar cellular targets and processes, and both trigger drastic changes in cell physiology. In microorganisms like yeast, optimal growth conditions result in downregulation of protective mechanisms since amplification of the population by maximal cell proliferation is from an evolutionary perspective a much better strategy for survival of the species than making fewer individual cells more stress tolerant. In multicellular organisms, mobilization of reserves under stress conditions is a plausible strategy for survival of the individual organism and thus for enhancing the potential for reproduction of the whole population.

The use of plasma membrane receptors is generally the best-known mechanism involved in the detection and response of cells to extracellular cues (Tell et al., 1978; Pierce et al., 2002). Also for detection of nutrients, plasma membrane receptors have been identified, as in the case of the glucose-sensing GPCR Gpr1 in yeast (Kraakman et al., 1999; Lemaire et al., 2004) and the taste receptors in mammals (Gilbertson et al., 2000; Lindemann, 2001). However, in the case of nutrients, other detection systems have also been discovered. These are based on the proteins that transport the nutrients into the cells and therefore seem to be more ancient sensor systems than the systems based on classical receptors. In the first case, transporter-like proteins have been identified in yeast that have lost the capacity to transport the substrate and have only retained a nutrient-sensing function. Snf3 and Rgt2 are high-affinity and low-affinity glucose sensors, respectively, (Ozcan et al., 1996) and Ssy1 is an amino acid sensor (Didion et al., 1998; Iraqui et al., 1999; Klasson et al., 1999; Ljungdahl, 2009). In both cases, these nutrient sensors regulate the expression of regular glucose or amino acid transporters, respectively. For that purpose, they make use of rather specific signal transduction pathways, for which to the best of our knowledge no similar pathways have been found in other classes of eukaryotic organisms. It appears most plausible that these nutrient sensor proteins first evolved from pure nutrient transporters into double transporter-receptor proteins, and then lost the transport capacity to become pure nutrient sensors, rather than that they first lost the transport capacity and then gained a receptor function.

The second case of the previously discussed nutrient transceptors that have retained their transport function is more striking in this respect because they also act on the PKA pathway and the same target systems as the glucose-sensing GPCR system that activates the cAMP-PKA signaling pathway (Conrad et al., 2014). Hence, they transmit the nutrient signal using the same signaling pathway as used by hormones in higher cells. As a result, all essential nutrients are able to cause through their transceptors the same drastic changes in cell physiology as observed upon hormone action in higher cells. For a microorganism like yeast rapid and appropriate adaptation to changes in the nutrient composition of the medium is critical for its survival in the fierce competition for food among microorganisms in the very rapidly changing conditions in microbial habitats in nature.

The precise mechanism by which these nutrient transceptors trigger activation of PKA is unclear. They do not seem to use cAMP as a second messenger. Nutrient transceptor mediated activation of PKA may represent an evolutionary ancestor of receptor-cAMP-PKA regulation. The presence of such nutrient transceptors in cells of higher eukaryotes would add a completely new dimension to regulation of hormone and other agonist receptor signaling by nutrient availability.

### Nutrient Transceptors as Potential Triggers for Nutrient Uptake by Endocytosis

Many nutrient transceptors undergo a substrate-induced endocytosis process similar to ligand-induced endocytosis of classical receptors. Such induced endocytosis processes result in the internalization of a small amount of extracellular fluid, which either could be recycled back to the medium through the multi-vesicular body recycling pathway for membrane proteins or could end up in the vacuole together with the endocytosed protein. Experimental evidence for different fates of endocytosed solutes has been obtained (Etxeberria et al., 2006). Little attention seems to have been paid up to now to the nutrients that are internalized into the cell together with the extracellular fluid during this endocytosis process. It seems plausible that endocytosed transporters continue to transfer their substrate from the endocytosed compartment to the cytosol during their stay in the endosomes, which implies that nutrient transceptors could also continue to activate downstream signaling pathways during their stay in the endosomes (Rubio-Texeira et al., 2012).

Interestingly, in plants endocytosis-based uptake of sucrose and also glucose has been reported. This raises the question as to how this could be initiated. One possibility would be a sugar transporter that acts as transceptor and triggers initiation of endocytosis resulting in delivery of sucrose directly to the vacuole (Etxeberria et al., 2005a; Baroja-Fernandez et al., 2006). Also for glucose, endocytosis-based delivery to the plant vacuole has been reported (Etxeberria et al., 2005b).

## Downstream Signaling Mechanisms

As described above, PKA controls cell growth and cell cycle progression by regulating transcription, translation, ribosome biogenesis and other important metabolic processes in response to nutrient availability (Conrad et al., 2014). Transceptors mediate activation of PKA when a missing essential nutrient is added again to stationary-phase cells deprived for that nutrient in a medium with fermentable sugar. It is important to realize that at that moment the transceptors for other nutrients for which the cells have not been starved are repressed and therefore unable to activate signaling to PKA. Only the transceptors of which the substrate is absent, and this can be a combination of nutrients, for instance nitrogen and iron, will be induced during the starvation period and therefore be ready to signal to PKA once the nutrient (or nutrients) are added back to the cells. Simultaneous starvation for two or more nutrients has not been investigated in detail, but we expect that if only one of the nutrients is added back to the cells, starvation for the remaining nutrients will dominate and any activation of PKA observed after addition of only one of the lacking nutrients would only be very short-lived since the cells unavoidably will remain in stationary phase due to the absence of the other nutrients. Only upon addition of all lacking nutrients, the cells will be able to exit stationary phase and start fermentative growth (Thevelein and de Winde, 1999). Multiple studies have focused on the possible molecular mechanisms involved in nutrient transceptor activation of the PKA pathway.

### Nutrient Transceptor Signaling Is Dependent on Glucose Sensing, But Itself Not Mediated by Ras-cAMP

As mentioned before, cAMP acts as a second messenger for PKA activation: when the level of cAMP in the cell increases, it binds to the PKA regulatory subunits causing their dissociation from the catalytic subunits, resulting in a higher percentage of free catalytic subunits that are active and can phosphorylate PKA targets (Santangelo, 2006). Addition of glucose or related rapidly fermentable sugars to yeast cells causes a rapid transient increase in the cAMP level, which is dependent on two converging mechanisms, the first uses the Ras proteins and the second the Galpha protein, Gpa2, as signal transducer (Mbonyi et al., 1988; Nakafuku et al., 1988; Colombo et al., 1998, 2004). Sugar transport and breakdown increases the level of fructose-1,6-bisphosphate, which stimulates the Ras proteins (Peeters et al., 2017). Hence, the first signal is based on sensing of intracellular glucose through its partial metabolism into a metabolic derivative that serves as metabolic messenger (Beullens et al., 1988). The yeast Ras proteins are not only potent activators of adenylate cyclase but are also required for basal adenylate cyclase activity. Without Ras activity the cells arrest in G1 and permanently enter the stationary G0 phase (Broach, 1991). Extracellular glucose is sensed by a GPCR, Gpr1, which activates adenylate cyclase through its Galpha protein Gpa2 (Kraakman et al., 1999). However, neither Gpr1 nor Gpa2 are essential for basal adenylate cyclase activity. Activation of cAMP synthesis by Gpr1-Gpa2 requires intracellular glucose phosphorylation (Rolland et al., 2000), likely through activation of Ras by fructose-1,6-bisphosphate (Peeters et al., 2017).

The presence of glucose is essential for transceptor-induced PKA signaling (Hirimburegama et al., 1992). It appears that the two glucose-sensing mechanisms are to some extent able to support transceptor-signaling separately (Pernambuco et al., 1996; Giots et al., 2003), while for glucose activation of cAMP synthesis, the intracellular glucose-phosphorylation-dependent mechanism is essential for extracellular glucose signaling through Gpr1 and Gpa2 (Beullens et al., 1988; Rolland et al., 2000). Glucose sensing may increase the level of free catalytic subunits to a certain extent, after which transceptor signaling may enhance their activity further. Glucose sensing through Gpr1 and Gpa2 is known to reduce the activity of the kelch-repeat proteins, which stimulate the binding of the PKA catalytic and regulatory subunits (Peeters et al., 2006, 2007; Budhwar et al., 2011). As a result, glucose sensing is able to increase the amount of free catalytic subunits in the presence of a constant cAMP level through inhibition of the kelch-repeat proteins. This may explain why the presence of glucose is required for nutrient transceptor signaling to PKA. Alternative mechanisms, however, cannot be excluded (see also further).

As opposed to glucose activation of the cAMP-PKA pathway, transceptor-induced activation does not trigger a significant increase in the cAMP level. This has been shown for Gap1, Mep2, Pho84, and Sul1,2, (Hirimburegama et al., 1992; Thevelein and de Winde, 1999; Kankipati et al., 2015). Moreover, experiments with yeast strains lacking the *BCY1* encoded regulatory subunits of PKA and having partially inactivating mutations in the *TPK* encoded catalytic subunits to avoid high constitutive activation of PKA, have shown that neither the regulatory subunits nor the Ras proteins are required for transceptor induced activation of PKA (Durnez et al., 1994). This has clearly shown that cAMP does not act as second messenger in nutrient transceptor signaling. However, this does not exclude that a basal level of cAMP may be required for transceptor signaling, since it may provide a sufficient amount of free catalytic subunits. This is suggested by the observation that although the *cdc25ts* mutant still shows nutrient transceptor signaling at the restrictive temperature of 37°C, displaying a cAMP level significantly below the wild type level under this condition, the extent of PKA activation is reduced compared to that at the permissive temperature in the same strain (Hirimburegama et al., 1992).

### Possible Involvement of Sch9 and Pkh1 in Transceptor Mediated PKA Signaling

The protein kinase Sch9 is the yeast ortholog of mammalian PKB/S6K, and is also homologous to the PKA catalytic subunits Tpk1-3 (Toda et al., 1988). Sch9 is involved in the regulation of cell size (Jorgensen et al., 2002), ribosome biogenesis (Huber et al., 2009) and the regulation of yeast life span (Fabrizio et al., 2001; Kaeberlein et al., 2005; Wanke et al., 2008). Sch9 is a major downstream substrate of TORC1, which plays an important role in intracellular nitrogen homeostasis (Urban et al., 2007). In addition to its role in the TOR pathway, Sch9 is required for amino acid induced activation of the PKA target trehalase by the Gap1 transceptor (Crauwels et al., 1997). However, deletion of Sch9 also increases basal PKA activity and thus strongly enhances the basal level of trehalase activity, which may interfere with transceptor-induced activation. Hence, it has remained unclear whether Sch9 is truly involved in the transceptor signaling pathway. Inhibition of the TOR pathway with rapamycin does not affect short-term activation of the PKA pathway through Gap1 and also for phosphate induced signaling to the PKA pathway by Pho84, TOR does not seem to be required (Conrad et al., 2017). Hence, at this moment the available data do not support involvement of the TOR-Sch9 axis in the nutrient transceptor signaling mechanism.

The yeast Pkh1-3 kinases are orthologs of mammalian PDK1 (Casamayor et al., 1999). Both Sch9 and Tpk1 can be phosphorylated by Pkh1 (Liu et al., 2005). The phosphorylation of Sch9 *in vivo* by Pkh1 is lost during nitrogen deprivation and reappears after nitrogen resupplementation, indicating that Sch9 phosphorylation by Pkh1 may in some way be connected to transceptor-mediated signaling. However, also in this case the available data do not support direct involvement in nutrient transceptor signaling (Voordeckers et al., 2011).

### Interaction of Transceptors With Downstream Regulatory Proteins

To identify possible signaling proteins involved in nutrient transceptor activation of the PKA pathway, a split-ubiquitin two-hybrid screen was performed to identify proteins physically interacting with the Gap1 amino acid transceptor and the Mep2 ammonium transceptor (Van Zeebroeck et al., 2011). Proteins involved in glycosylation, the secretory pathway, sphingolipid biosynthesis, cell wall biosynthesis and other processes were identified. Deletion of a significant number affected both amino acid transport and signaling by Gap1 and/or Mep2 indicating that the proteins were in some way involved in Gap1 and/or Mep2 secretion or their functioning at the plasma membrane. Transport and signaling were differentially affected in specific deletion strains, clearly separating the two functions of the transceptors and confirming that signaling does not require transport. However, these findings have not yet led to a breakthrough in our understanding of the mechanism by which the nutrient transceptors signal to the PKA pathway.

Interestingly, Sui2 was identified as a Gap1 binding partner in this screen. Sui2 is the alpha subunit of eukaryotic initiation factor 2 (eIF2), a G-protein that plays an essential role in the initiation of protein synthesis and cell growth (Sonenberg and Hinnebusch, 2009). The close relationship between nutrient transceptor signaling and resumption of growth in nutrient-deprived cells, suggests that there might be a mechanistic connection between nutrient transceptor signaling and nutrient activation of eIF2 to initiate protein synthesis. Amino acid deprivation is well known to enhance the level of uncharged tRNAs, which triggers activation of the Gcn2 kinase. Gcn2-mediated phosphorylation of the eIF2 alpha subunit inhibits its guanine nucleotide exchange factor eIF2B, leading to downregulation of bulk protein synthesis and Gcn4-mediated expression of specific nitrogen starvation induced genes involved in amino acid biosynthesis and uptake (Baird and Wek, 2012). Deletion of *GCN2* strongly enhances Gap1 transceptor signaling in a *leu2* auxotrophic mutant, but not in a wild type strain, making direct involvement of Gcn2 and/or eIF2 in Gap1 transceptor signaling unclear (Conrad et al., 2017).

### A Hypothesis for Transceptor-Mediated Downregulation and Re-Activation of PKA

As previously explained, re-addition of the nutrient for which the cells have been starved triggers transceptor endocytosis and degradation in the vacuole. Hence, transceptor signaling to PKA has to be transient. In spite of this, the cells maintain a high PKA phenotype throughout exponential fermentable growth, conditions under which all starvation-induced transceptors remain repressed. This may suggest that fermentable sugar like glucose continuously activates PKA through the cAMP pathway during fermentative growth and that starvation-induced downregulation of PKA is caused in a cAMP-independent way by the transceptors. One straightforward and plausible hypothesis could be that the free catalytic subunits of PKA bind to the transceptor(s) and remain in this way sequestrated from their targets as long as the cells remain starved for at least one essential nutrient. This would result in the typical downregulation of the whole range of PKA targets, which is observed in all stationary-phase cells. In this case, the many different transceptors would only need a common intracellular binding site for the catalytic subunits of PKA, which would be exposed when the substrate of the transceptor is absent. When the substrate is added and transported by the transceptor, the latter changes its conformation and releases the catalytic subunits of PKA, triggering the typical sudden burst in PKA activity. This hypothesis can explain in a straightforward manner why so many different nutrients can trigger the same downregulation and re-activation of PKA through their own specific transceptor using basically the same mechanism. The glucose requirement of nutrient transceptor signaling can then be explained by the requirement of maintaining an adequate cAMP level and thus also an adequate amount of free catalytic subunits of PKA. Moreover, the presence of glucose also causes inactivation of the kelch-repeat proteins Krh1,2, which strengthen the binding of the catalytic and regulatory subunits. Their inactivation thus increases the amount of free catalytic subunits even in the presence of a constant cAMP level (Peeters et al., 2006, 2007; Budhwar et al., 2011). In the absence of glucose any catalytic subunit released by the transceptor would thus rapidly be inactivated by free regulatory subunits, or even the amount of catalytic subunits bound to the transceptor might be too low to detect any significant transceptor signaling. The same explanation may hold for cells growing respiratively in complete medium on a non-fermentable carbon source, in which PKA activity is also known to be significantly lower than during fermentative growth on glucose. In this case, glucose is absent and the Krh proteins are active, stimulating the binding between catalytic and regulatory subunits of PKA and thus reducing its activity at constant cAMP level. This effect may be reinforced further by sequestration of catalytic subunits through binding to high-affinity glucose transporters, known to be derepressed in the absence of glucose, and thus functioning as putative transceptors. Hence, in spite of the fact that fermentatively and respiratively grown yeast cells have a very similar cAMP level (Eraso and Gancedo, 1984; Ma et al., 1997; Thevelein and de Winde, 1999), these mechanisms can explain why their PKA activity is very different.

## Evolutionary Origin of Transceptors

In virtually all eukaryotic cell types, transporters and receptors are found in the plasma membrane. It seems plausible that nutrient transporters originated first in evolution since for any unicellular organism nutrient import is essential for survival and proliferation. Sensing and signaling by receptor proteins appears as a more sophisticated property generated later in evolution. It is unclear how receptors originated in evolution, but the close similarity between neurotransmitters and nutrients has led many years ago already to the suggestion that receptors might originate from nutrient transporters (Boyd, 1979). The latter are able to import nutrients into the cell and are therefore by definition also able to detect nutrient molecules in the environment. Therefore, nutrient transporters seem well suited to function as evolutionary precursors for receptors.

**FIGURE 2:**
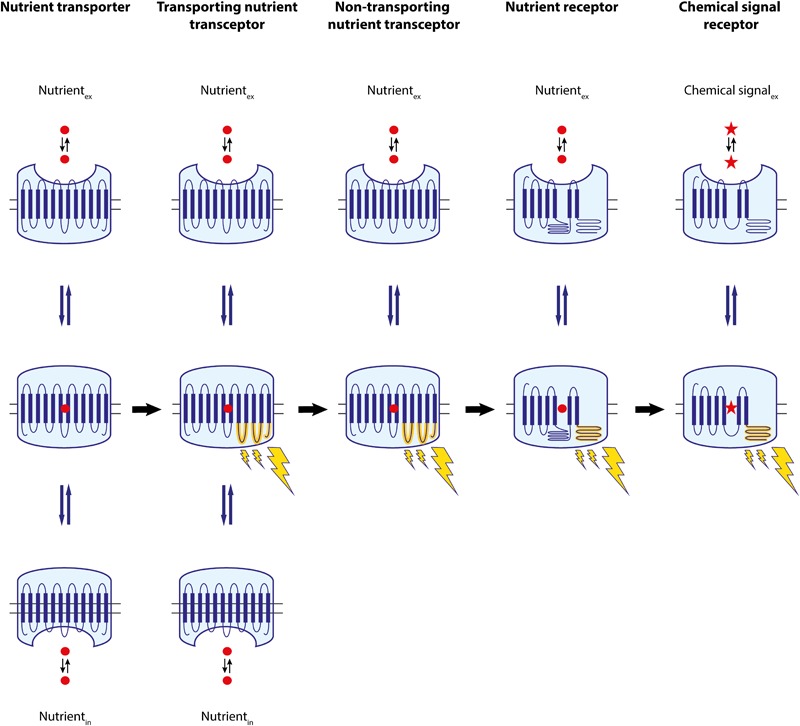
Overview of the proposed development of receptors from nutrient transporters during evolution. Initially cells only had nutrient transporters, which can transition between an outward- and an inward-facing conformation through one or likely more intermediate conformations. We suggest that one of the intermediate conformations gained the capacity to interact with an intracellular (regulatory) protein, modifying the nutrient transporter into a nutrient transceptor, which still retained its transport capacity. Subsequently, some of these transceptors lost their transport capacity, evolving into a pure transporter-like nutrient sensor or a non-transporting nutrient transceptor. We suggest that some of these proteins underwent modifications in their structure and evolved into nutrient receptors, losing their prominent sequence similarity with the original transporters and establishing a new family of receptors. Further evolution of these proteins generated a wide diversity of related receptors for a great variety of chemical signals.

The discovery in yeast of nutrient transporters that also display a nutrient signaling function and transporter-like proteins that have lost their transport capacity and function as pure nutrient sensors provides support for this concept (Thevelein and Voordeckers, 2009). It appears plausible that during evolution certain nutrient transporters gained the capacity to interact with an intracellular protein signaling the presence of the nutrient to the intracellular machinery (**Figure 2**). This could provide an evolutionary advantage to a microorganism in allowing it to adapt its metabolism, gene expression machinery and/or other cellular properties more rapidly, i.e., before the nutrient has been able to affect these properties through its metabolism, i.e., by increasing energy levels, reducing power or availability of precursors. Nutrient transporters undergo multiple conformational changes during the transport cycle. Hence, it is not difficult to envisage that a specific conformation triggered by transport of the substrate would allow the transporter to interact with an intracellular protein, whereas the transporter in its basic conformation would not be able to do so. The yeast high-affinity transporters that are strongly induced during deprivation of their substrate, Gap1, Mep2, Pho84, Sul1,2, Ftr1, and Zrt1, and that all signal to the PKA pathway, are prominent examples of such transporters that gained an additional signaling function to rapidly affect a pleiotropic range of cellular properties upon detection of their nutrient substrate. It appears likely that loss of the transport function in such transporter-receptors has led to the transporter-like proteins with a pure nutrient sensing function (**Figure 2**). These proteins have retained the capacity to bind the nutrient substrate, possibly allow it to enter to some extent the transportation channel, and as a result switch the transporter conformation into the signaling conformation. Further transport of the nutrient toward release into the cytosol would have become defunct. The yeast Snf3, Rgt2, and Ssy1 proteins are prominent examples of such nutrient sensors. The demonstration that the mammalian SGLT3 non-transporting glucose transceptor can be converted into an actively transporting carrier by a single amino acid change in the glucose-binding domain provides strong support that the transition from transporting transceptor to non-transporting receptor would be a simple evolutionary step (Bianchi and Diez-Sampedro, 2010).

We may consider all transporter-related proteins with a nutrient signaling function as transporter-receptors or transceptors. Hence, there are transporting and non-transporting transceptors. The distinction between the two categories may not be sharp. It is quite likely that eukaryotic transceptors with a primary signaling function may be discovered that have retained residual transport activity. Actually, such a protein has been discovered already in *Escherichia coli*. The *UhpT* gene encodes a glucose-6-phosphate transporter, while the adjacent isogene encodes a glucose-6-phosphate sensor, but the latter has retained residual glucose-6-phosphate transport activity (Schwoppe et al., 2003). Intermediate forms between transporter-related nutrient sensors and nutrient receptors may also be discovered. Actually, the yeast glucose-sensing GPCR, Gpr1, has an unusually long third intracellular loop with seemingly superfluous asparagine repeats, and interacts with a Galpha protein which functions without a genuine beta/gamma subunit, supporting its primitive evolutionary character (Thevelein and de Winde, 1999). Conversion of the third intracellular loop into five transmembrane domains with retention of the glucose-sensing and signaling function of the Gpr1 receptor, would constitute an interesting argument for an evolutionary origin of Gpr1 from a glucose transporter. Interestingly, a fragment with a similar sequence has been identified in organic ion transporters able to bind odorants and in genuine ORs of the GPCR family in nasal epithelial cells. This fragment is located in regions believed to be important for ligand/substrate preference and recognition in both classes of proteins, raising the possibility that it may be part of a potential common ligand/substrate recognition site. Coexpression of these proteins in olfactory tissues suggests that they may function in concert (Wu et al., 2015). These data raise the question as to whether these organic ion transporters may function as transceptors. Further diversification of nutrient receptors may have led to the great variety of chemical signal receptors that we know today, for instance in the huge family of GPCRs (**Figure 2**).

The similarity of transporting transceptors and classical receptors in undergoing substrate/ligand-induced internalization also extends to the non-transporting transceptors. The low-affinity glucose sensor Rgt2 remains stable only in high-glucose grown cells, and the high-affinity glucose sensor Snf3 is stable only in cells grown in media with a low glucose level. Both proteins undergo ubiquitination-induced internalization and degradation in the vacuole under adverse conditions. In addition, constitutively active, signaling forms of the glucose sensors do not undergo endocytosis, whereas signaling defective sensors are constitutively targeted for degradation, suggesting that the stability of the glucose sensors may be associated with their ability to sense glucose (Roy and Kim, 2014).

## Identification Of Nutrient Transceptors In Other Eukaryotes

Subsequent to the discovery of transporting and non-transporting nutrient transceptors in yeast, many examples of nutrient transceptors have been found or suggested in other eukaryotes. As in yeast, most of these transceptors have been discovered in connection with regulatory responses to deprivation or to resupplementation of the substrate of the transceptor. Hence, these transceptors generally function in specific processes controlled by the substrate of the transceptor and often related more or less directly to the metabolism and/or the uptake of the substrate. For several of these transceptors there is evidence for control of multiple downstream processes and in some cases it has been shown that separate signaling events are involved. There are no clear examples yet in other eukaryotes of transceptors for different nutrients that control the same signaling pathway, as in the case of the yeast transceptors that trigger activation of the PKA pathway. In general, disconnection between uptake activity of a transporter and/or metabolism of its nutrient substrate and one or more regulatory responses triggered by the nutrient has led to awareness that the transporter might act as a sensor. Convincing experimental demonstration generally requires further detailed genetic modification of the transporter to separate transport and sensing more clearly to show that it truly functions as a transceptor.

### Other Yeast Species and Filamentous Fungi

Nutrient transceptors have been identified in other yeast species as well as in filamentous fungi. Similar to the ammonium transceptor Mep2 in *S. cerevisiae*, the Mep family members Ump2 in *Ustilago maydis* (Smith et al., 2003; Paul et al., 2014), MepB in *Fusarium fujikuroi* (Teichert et al., 2008), Amt1 in *Hebeloma cylindrosporum* (Javelle et al., 2003), MepB in *Colletotrichum gloeosporioides* (Shnaiderman et al., 2013), Amt1,2 in *Cryptococcus neoformans* (Rutherford et al., 2008; Lee et al., 2012) and Mep2 in *Candida albicans* (Biswas and Morschhauser, 2005) have been shown or suggested to function also as ammonium sensors. Their precise mode of action generally remains unknown. The transceptors and pathways involved also seem to be different in some instances, for instance in *Fusarium fujikuroi* MepB was essential for repression of nitrogen regulated genes, as opposed to MepA and MepC, whereas only MepA and MepC, and not MepB, were able to complement the *S. cerevisiae mep2*Δ mutant for pseudohyphal growth induction (Teichert et al., 2008). On the other hand, ammonium uptake through MepB in *C. gloeosporioides* also appears to activate the PKA pathway (Shnaiderman et al., 2013), as in *S. cerevisiae*.

While in baker’s yeast, no transporting transceptors for glucose or other sugars have yet been identified, examples of non-transporting hexose transceptors in other fungi that are homologous with the yeast Snf3 or Rgt2 glucose sensors have been reported. Examples are Rag4 in *Kluyveromyces lactis* (Betina et al., 2001), Hgt4 in *C. albicans* (Brown et al., 2006), Gcr1 and Hxs1 in *Hansenula polymorpha* (Stasyk et al., 2004, 2008), Rco3 and Hgt-1, Hgt-2 in *Neurospora crassa* (Madi et al., 1997; Wang B. et al., 2017) and Hxt1 in *U. maydis* (Schuler et al., 2015). A homolog of the yeast amino acid sensor Ssy1 has been reported in *C. albicans* (Brega et al., 2004). No clear homologs of the yeast non-transporting transceptors Snf3, Rgt2, and Ssy1 have been identified in higher eukaryotes.

In *N. crassa*, two cellodextrin transporters, Cdt1,2 have been shown to contribute to cellobiose and cellodextrin sensing for induction of cellulolytic enzymes (Znameroski et al., 2014). In addition, cellodextrin transporter-like protein 1 (CLP1; NCU05853) was shown to be a critical component of the cellulase induction pathway and also regulates the expression of the cellodextrin transporters Cdt1,2. Interestingly, CLP1 lacks cellodextrin transport activity and thus seems to function in a similar way as the non-transporting glucose and amino acid transceptors in the yeast *S. cerevisiae* (Cai et al., 2015). The Hgt-1, Hgt-2 glucose transporters are involved in carbon catabolite repression of the same cellulase expression system in *N. crassa* (Wang B. et al., 2017). Hence, multiple transceptors seem to act on the same target system.

### Animals and Humans

Apart from yeasts and fungi, transceptors have also been identified in higher eukaryotes. One of the best documented examples is the mammalian sodium-dependent neutral amino acid transporter 2, SNAT2, which is strongly induced by amino acid deprivation (Hyde et al., 2007; Hundal and Taylor, 2009; Taylor, 2014), just like the yeast Gap1 amino acid transceptor. When sufficient amino acids are present, SNAT2 represses through multiple mechanisms its own expression in a transport-dependent way, hence SNAT2 functions as a transceptor. Interestingly, the amino acid analog *N*-methyl-aminoisobutyric acid prevents SNAT2 expression and affects cell size without effect on total cellular protein content, although it is transported only very slowly by SNAT2 and acts as a competitive inhibitor of amino acid uptake (Pinilla et al., 2011). This is similar to the non-transported signaling agonists of the yeast amino acid transceptor Gap1 (Van Zeebroeck et al., 2009). These data suggest that screening a collection of substrate analogs in order to identify non-transported signaling agonists is a powerful strategy to obtain strong evidence for receptor functionality in a nutrient transporter. In *Drosophila* the amino acid transporter PATH, which is related to mammalian proton-assisted amino acid transporters (PATs), was shown to control growth via a mechanism that does not require bulk transport of amino acids into the cell (Goberdhan et al., 2005).

The TORC1 complex has been identified as a nitrogen-sensing system in yeast and mammalian cells (Kim and Guan, 2011; Loewith and Hall, 2011). Mammalian mTORC1 moves from the cytosol to the lysosomal membrane upon addition of amino acids to cells deprived of amino acids (Sancak et al., 2010). At the lysosomal membrane, TORC1 contacts the lysosomal arginine transporter, SLC38A9, which has been proposed to act as a transceptor for sensing the arginine level in the lysosome and regulating TORC1 in response (Jung et al., 2015; Rebsamen et al., 2015; Wang et al., 2015). Two other mammalian amino acid transporters have been suggested to function as transceptors in connection with the functioning of the TORC1 complex, the plasma membrane L-type amino acid transporter LAT1 and the lysosomal proton-assisted amino acid transporter PAT1/SLC36A1 (reviewed by (Ogmundsdottir et al., 2012; Zheng et al., 2016). Recent work has shown that there exists apparently a relationship between the expression response of amino acid transceptors as a function of the dietary protein level in skeletal muscle tissue of different fiber type. This may suggest a relationship between transceptor response and growth performance (Li Y.H. et al., 2017). In *Drosophila* (Colombani et al., 2003; Bjordal et al., 2014) and the mosquito *Aedes aegypti* (Boudko et al., 2015) the amino acid transporter Slimfast has been suggested to function as a transceptor. The same is true for the SNF-5 amino acid transporter in the nematode *Caenorhabditis elegans* (Metzler et al., 2013). However, in most of these cases, the evidence for a transceptor function is still circumstantial.

The human SGLT3/SLC5A4 glucose transporter homolog, expressed a.o. in cholinergic neurons and skeletal muscle, was suggested to function as a glucose sensor since no glucose uptake activity could be demonstrated for the protein upon expression in *Xenopus oocytes*, although glucose caused a specific, phlorizin-sensitive, Na^+^-dependent depolarization of the membrane potential (Diez-Sampedro et al., 2003). Interestingly, mutagenesis of a single amino acid residue, Glu457, in SGLT3 into the corresponding residue Gln of the SGLT1 transporter converted SGLT3 into an active glucose transporter with very similar kinetic characteristics as SGLT1. Expression of the wild type and mutant forms of SGLT3 in *C. elegans* sensory neurons conferred glucose-sensing capacity in an SGLT3-dependent manner, providing evidence that the protein can function *in vivo* as a glucose sensor (Bianchi and Diez-Sampedro, 2010).

The mammalian GLUT1 transporter has been proposed to function as a glucose transceptor for activation of extracellular signal-regulated kinase (ERK) by direct interaction with proline-rich tyrosine kinase-2 (PYK2) (Bandyopadhyay et al., 2000). Loss of sugar sensing through modification of GLUT2 in transgenic mice affected multiple aspects of glucose homeostasis, which led to the suggestion that GLUT2 might act as a transceptor for glucose-regulated processes (Stolarczyk et al., 2007). Specific mutations in human GLUT2 affected insulin secretion and/or development in beta cells in the absence of glucose, suggesting that GLUT2 triggers as transceptor a signaling pathway independently of glucose transport and metabolism (Michau et al., 2013). Specific mutations in GLUT2 have been associated with several human diseases but in most cases it remains unclear whether this is due to impaired glucose uptake or any of its metabolic consequences, or whether it reflects involvement of GLUT2 as a glucose transceptor (Thorens, 2015).

Expression of a transport deficient mutant form of the human CNT1 nucleoside transporter in pancreatic cancer cells affected multiple processes in a similar way as expression of the wild type CNT1 transporter, which led to the suggestion that it may function as a transceptor (Perez-Torras et al., 2013).

In mammalian cells, it was recently found that OATs and ORs may function in concert via their ability to respond to odorant ligands. Moreover, OATs possess an evolutionarily conserved fragment, possibly playing a role in substrate recognition, that is also present in ORs of the GPCR family (Wu et al., 2015). Interestingly, in neuronal astrocytes a similar situation has been reported. Evidence was provided for involvement of both glutamate receptors and transporters in glutamate signaling for activation of an intracellular mitogen-activated protein kinase (MAPK) cascade mediating the cellular responses to the neurotransmitter. A major argument was provided with transported analogs, like D-aspartate and small molecule inhibitors, that elicited the same response as glutamate (Abe and Saito, 2001). More recent work has extended these findings to protein synthesis stimulation in glia cells (Martinez-Lozada et al., 2011; Maria Lopez-Colome et al., 2012; Martinez-Lozada and Ortega, 2015).

### Plants

Also in plants strong evidence has been obtained for the existence of nutrient transceptors. The major nitrate transporter NRT1.1 is the best characterized nutrient transceptor in plants and mediates several nitrate signaling responses. Specific amino acid modifications have allowed to uncouple transport and signaling (Krouk et al., 2006; Remans et al., 2006; Ho et al., 2009; Gojon et al., 2011). The NRT1.1 transceptor in *Arabidopsis thaliana* affects different regulatory modules in response to nitrate. It is apparently able to activate four separate signaling mechanisms and specific mutagenesis resulted in differential effects on the downstream targets. NRT1.1 affects nitrate uptake and metabolism at the gene expression level and also controls root morphogenesis and other processes (Walch-Liu and Forde, 2008; Bouguyon et al., 2015; Giehl and von Wiren, 2015; Li Z. et al., 2017).

Phosphate sensing has been studied intensively in Arbuscular Mycorrhizal Symbiosis, in which endosymbiont fungi provide nutrients, in particular phosphate, to the host plant. Strong evidence has been obtained for a phosphate transceptor function of the high-affinity phosphate transporter GigmPT in the fungal symbiont *Gigaspora margarita*. It functions apparently in a manner very similar to the *S. cerevisiae* Pho84 phosphate transceptor, using a similar phosphate binding site and also connecting to the PKA signaling pathway (Xie et al., 2016). Arbuscular Mycorrhizal Symbiosis induces the expression of multiple phosphate transporters in plants, of which the expression is actually essential for development and maintenance of the mycorrhizal symbiont (Javot et al., 2007; Johri et al., 2015; Mlodzinska and Zboinska, 2016; Wang D. et al., 2017). Some of these transporters may play a role as transceptors. Mutation or downregulation of *OsPHT1;13* by RNAi in *Oryza sativa* causes strong reduction of fungal colonization and arbuscule development, but apparently without effect on phosphate acquisition, and the OsPHT1;13 protein does not seem to possess phosphate transport capacity (Yang et al., 2012). Comparable sensor function was shown for AsPHT1;1 (AsPT1) from *Astragalus sinicus*, but this protein still exhibits transporting activity (Xie et al., 2013).

Phosphate availability also strongly affects root morphology and branching in plants irrespective of the presence of mycorrhizal symbionts (Mlodzinska and Zboinska, 2016). For these responses the PT4 phosphate transporters in *Lotus japonicus* and *Medicago truncatula* were suggested to act as phosphate transceptors (Volpe et al., 2016).

The ammonium transporter AMT1;3 has been shown to act as a transceptor in the plant *L. japonicus* for ammonium modulation of root development (Lima et al., 2010; Rogato et al., 2010a,b). Expression of the *A. thaliana* AtAmt1;1, AtAmt1;2, AtAmt1;3, and AtAmt2 ammonium transporters in the *S. cerevisiae mep1*Δ *mep2*Δ *mep3*Δ strain sustained transport and trehalase activation to different extents (Van Nuland et al., 2006), suggesting that they may also function as transceptors in the plant. The sulfate transporter SULTR1;2 in *A. thaliana* was shown to control expression of the sulfur deficiency-activated *BGLU28* gene encoding beta-glucosidase 28 independent of intracellular accumulation of sulfate or derived metabolites, leading to the suggestion that it may function as a sulfate transceptor (Zhang et al., 2014). A glycine residue possibly conserved in all eukaryotic sulfate transporters might have universal importance for both transport and signaling (Zheng et al., 2014).

## Conclusion

The early demonstration that the yeast *S. cerevisiae* contained transporter-like proteins that had lost their transport function and functioned purely as nutrient sensors for glucose or amino acids was easily accepted by the scientific community. The demonstration that the classical *S. cerevisiae* Gap1 amino acid transporter had an additional receptor function to signal to the PKA pathway was also a new concept but more challenging to establish convincingly. First, because of the difficulty to demonstrate unequivocally that the signaling was triggered by the transporter itself and not by the amino acid substrate entering the cytosol. This issue has remained the major challenge in the field of nutrient transceptors: to clearly separate the receptor signaling function of the transceptor from its substrate transport function. Site-directed mutagenesis to affect specifically only one of the two functions is a powerful approach, but also the use of non-transported substrate analogs that are able to activate the receptor function as ligands has been very powerful. Second, the claim that a simple nutrient was able to activate through a seemingly simple housekeeping nutrient transporter a sophisticated signaling pathway like the PKA pathway, normally activated by dedicated primary messengers functioning as intercellular signaling molecules and mediated by dedicated and complex receptor protein systems, was apparently difficult to rationalize.

We have coined the term ‘transceptor’ in our TIBS review in Holsbeeks et al. (2004). Since then the term transceptor has slowly spread in the literature and more than 50 papers have already appeared with this term in title or abstract. More papers have dealt with the same issue without using the term transceptor. The difficulty of identifying transceptors unambiguously and the challenges generally involved in analysis of membrane proteins, in particular their interaction with intracellular signaling proteins, may explain the seemingly slow progress. On the other hand, the data assembled in this review suggest that transceptors likely exist for all nutrients, both macro- and micro-nutrients, that they may interact with multiple separate signaling modules and that they show a very broad range of downstream targets, from highly specific controls on the expression and/or activity of the transceptor itself to signaling pathways affecting metabolic, stress response, morphogenetic and many other cellular processes.

The expanding world of transceptors seems to suggest that they may represent a cellular control mechanism as broad and as powerful as that of the regular receptors. From an evolutionary viewpoint this may not be surprising since for the first cellular organisms in evolution detection and response to nutrients was undoubtedly paramount for their survival in the competition for scarce resources. In higher organisms, the receptor function seems to be more dominant for control of life processes, with nutrients apparently being largely confined to playing a purely supporting role as providers of energy and building blocks. Because of the dominance of hormone and growth factor regulation by a multitude of receptor systems, and the difficulty of separating regulatory functions of nutrients from their supporting functions in metabolism, regulation by nutrient transceptors may be much more widespread than generally anticipated and thus largely overlooked up to now. It might even turn out that most, if not all nutrient transporters, interact in some way with at least one intracellular regulatory protein and may thus function as transceptors.

The many similarities between genuine receptor and nutrient transceptor regulation and functioning suggest that receptors are derived in evolution from nutrient transporters with nutrient transceptors as one of the possible transition forms. It also suggests that the many peculiarities discovered for regular receptors, such as biased agonist signaling and multiple binding sites, may also exist for nutrient transceptors.

As in the past, discrepancy between transport activity of a nutrient transporter and the extent of substrate-induced effects mediated by the transporter on downstream cellular processes will likely continue to serve as a strong lead for the discovery of new transceptors.

The major current challenges in the field include the identification of the signal transduction pathways activated (or inhibited) by nutrient transceptors, in particular the signaling proteins that directly interact with the nutrient transceptors, the identification of other examples of the same cellular process affected by multiple transceptors for different nutrients, the identification of similar signaling domains and downstream signaling pathways in transceptors from multiple distantly related species and elucidation of the connection between substrate binding, transport and signaling in a transceptor.

## Author Contributions

All authors listed have made a substantial, direct and intellectual contribution to the work, and approved it for publication.

## Conflict of Interest Statement

The authors declare that the research was conducted in the absence of any commercial or financial relationships that could be construed as a potential conflict of interest.

## Abbreviations

eIF: eukaryotic initiation factor
FGM: fermentable growth medium
Gly3P: glycerol-3-phosphate
GPCR: G-protein coupled receptor
OAT: organic anion transporter
OR: odorant receptor
PDK1: 3-phosphoinositide-dependent protein kinase-1
PKA: protein kinase A
TORC1: target of rapamycin complex 1
